# Financial inclusion and environmental pollution in sub-Saharan Africa: moderating effects of economic growth and renewable energy

**DOI:** 10.1007/s11356-024-34785-7

**Published:** 2024-09-04

**Authors:** Rabie Said

**Affiliations:** La Trobe Business School, La Trobe University, Melbourne, Australia

**Keywords:** CO_2_ emissions, Financial inclusion, Renewable energy, Economic growth, Sub-Saharan Africa, Panel data

## Abstract

A thriving literature exists about the role of financial inclusion in socio-economic development. Nevertheless, the environmental effects of financial inclusion are largely unknown in the literature, especially in sub-Saharan African countries. Therefore, this study explores the association between financial inclusion and CO_2_ emissions utilizing data from 23 sub-Saharan Africa for the period 2004–2019. Based on different estimation methods such as dynamic ordinary least squares (DOLS), fully modified ordinary least squares (FMOLS), canonical correlation regression (CCR), and an instrumental variable generalized-method of moment (IV-GMM), the results show that financial inclusion is responsible for a substantial increase in CO_2_ emissions. In addition, financial inclusion moderates economic growth, resulting in higher CO_2_ emissions. Alternatively, financial inclusion moderates renewable energy use to lower CO_2_ emissions. The outcomes also verify the presence of the Environmental Kuznets Curve hypothesis (EKC). This study proposes uniting financial inclusion and environmental policies as a strategy for reducing CO_2_ emissions in sub-Saharan Africa.

## Introduction

Climate change is today’s most contentious environmental matter, which has gained the attention of policymakers (Amin et al. 2022; Said and Acheampong 2024). The increasing emphasis on greenhouse gases in the atmosphere has serious consequences for both economic and human development (Tamazian et al. 2009; Shahbaz et al. 2022; Hussain et al. 2023). In spite of attempts to diminish CO_2_ emissions, international emissions have been rising. According to the World Bank (2023), worldwide CO_2_ emissions reached to more than 34 million kilotons (kt) in 2019 compared to around 29 million (kt) in 2009. This enormous increase in CO_2_ emissions contradicts the Paris consent on climate change to alleviate CO_2_ emissions.

It is undeniable that economic growth improves living standards, but it is also harmful to the environment (Acheampong 2019; Mehmood 2022; Naseem et al. 2024; Işık et al. 2024). Following the work of Grossman and Krueger (1995), numerous studies have assessed the link between economic growth and environmental pollution, notably recognized as the Environmental Kuznets curve (EKC). The EKC hypothesis recommends that economic growth and the environment have an inverted U-shape relationship. Thus, during the early phases of growth, environmental pollution increases, but after a certain threshold of growth, it decreases. Empirical findings on the EKC hypothesis have been inconsistent. Some authors such as Farhani and Ozturk (2015), Acheampong (2019), Tenaw and Beyene (2021), and Naseem et al. (2024) state that CO_2_ emissions are continuously rising with economic growth; thus, the EKC hypothesis is not supportable in this case. In contrast, other works have maintained the presence of the EKC postulate (see, e.g., Stern 2004; Tamazian et al. 2009; Omri et al. 2015; Sapkota and Bastola 2017; Aydoğan and Vardar 2020; Jebabli et al. 2023; Raza and Tang 2024).

Theoretically, the impact of financial inclusion on the environment remains debatable. Some authors are with the standpoint that financial inclusion enhances the quality of the environment through diminishing CO_2_ emissions. Jensen (1996), for example, argues that the financial sector plays a considerable role in lowering energy emissions through encouraging technological advancements in the energy supply to minimize environmental pollution. In addition, as Tamazian et al. (2009) point out, financial services are the source of greater levels of research and development, which ultimately benefit the environment. Other scholars, however, argue that financial development can have detrimental effects on environmental quality, which contradicts these desirable impacts of financial services on the environmental quality. Sadorsky (2011), as an instance, argues financial development leads to more energy consumption and environmental degradation related to energy-intensive consumer items like refrigerators, cars, and big houses. Furthermore, financial inclusion could boost CO_2_ emissions through stimulating industrial manufacturing and utilizing energy-intensive consumable items (Le et al. 2020).

Despite renewable energy’s favorable environmental effects, its role in reducing CO_2_ emissions remains controversial. Some scholars support the opinion that renewable energy upgrades the environment. For example, Huang et al. (2021) contend that renewable energy use has a beneficial influence on the environment by creating a minimal carbon footprint in comparison with fossil fuels consumption. They also contend that coal use results in 3.6 pounds of CO_2_ E/kWh in comparison with 0.04 pounds produced through wind. Moreover, renewable energy leads to the increasing demand for energy use driven by economic growth (Rasoulinezhad et al. 2020). Contrarily, other group of scholars claim that renewable energy worsens the environment by rising CO_2_ emissions. Nguyen and Kakinaka (2019) find that renewable energy exerts a positive influence on CO_2_ emissions in developing nations. The authors stress that in developing countries, a lack of modern and affordable forms of energy decreases economic productivity and income-generating opportunities, so they cannot afford to promote cleaner energy. Furthermore, renewable energy projects may need highly sophisticated technology and relatively high costs, which can be quite burdensome for national budgets.

Till date, few papers have assessed the effect of financial inclusion on CO_2_ emissions and provided the contradictions in the literature; Acheampong (2019), Saidi and Omri (2020), and Said and Acheampong (2024) contend that further empirical studies should be conducted to resolve these inconsistent findings. Thus, investigations of financial inclusion and the environment are still in the early phases, and additional studies are needed to adequately comprehend how financial inclusion affects the environment. In addition, little research has been conducted to assess how financial inclusion moderates economic growth and renewable energy to influence on CO_2_ emissions. As a final point, scarcely is shown about the influence of financial inclusion on CO_2_ emissions in sub-Saharan Africa. Based on literature disagreements, this paper explores the direct and indirect impacts of financial inclusion on CO_2_ emissions in 23 sub-Saharan African nations between 2004 and 2019.

**Fig. 1:**
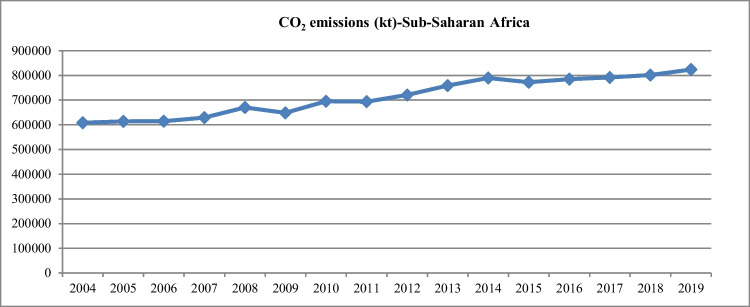
CO_2_ emissions (kt)-sub-Saharan Africa. Source: The World Bank (2023)

This study focuses on the sub-Saharan African region since the region is the lowest contributor to worldwide CO_2_ emissions. However, sub-Saharan Africa has experienced a continuous growth in CO_2_ emissions over the past two decades. For example, according to the World Bank (2023), CO_2_ emissions increased from 608,000 (kt) in 2004 to 789,000 (kt) in 2014 and further to about 824,000 (kt) in 2019 (see Fig. 1).

**Fig. 2:**
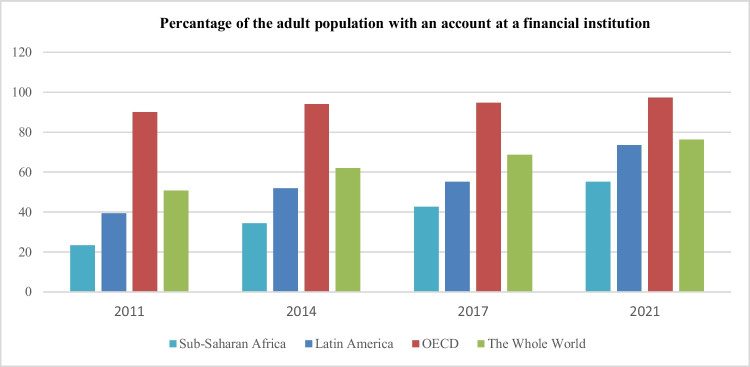
Percentage of the adult population with an account at a financial institution. Source: Global Findex database 2022

The region has also experienced a significant improvement in financial inclusion. Despite improvements in the percentage of adults with bank accounts in sub-Saharan Africa between 2011 and 2021, only 55% of adults in this region had bank accounts in 2021. In this region, account ownership does not only lag behind high-income countries and the world average but also behind other developing nations (see Fig. 2). Therefore, it is crucial to understand how financial inclusion impacts CO_2_ emissions in sub-Saharan Africa to develop climate change policies and for sustainable development in the region.

As of now, very few studies have simultaneously incorporated financial inclusion, economic growth, and renewable energy in their model. Therefore, this study evaluates whether financial inclusion, economic growth, and renewable energy simultaneously affect CO_2_ emissions. In addition, it examines whether renewable energy and economic growth activities moderate the relationship between financial inclusion and environmental pollution, which has largely been neglected in the previous studies. Consequently, this paper addresses three issues that are lacking in the past research. (1) How does financial inclusion affect the environment in the sub-Saharan African region? (2) Does economic growth moderate the connection between financial inclusion and CO_2_ emissions? (3) Do renewable energy sources moderate the linkage between financial inclusion CO_2_ emissions in the region?

This study extends and contributes to knowledge by the following ways. First, it examines the direct relationship between financial inclusion and CO_2_ emissions for 23 countries in sub-Saharan Africa between 2004 and 2019. Second, it illustrates indirect effects of financial inclusion on CO_2_ emissions. Hence, in contrast to previous studies, the present study extends the literature by looking at how financial inclusion moderates the impact of economic growth and renewable energy on CO_2_ emissions. As a final point, to accomplish consistent and robust empirical findings, this study utilizes different techniques such as dynamic ordinary least squares (DOLS), fully modified ordinary least squares (FMOLS), canonical correlation regression (CCR), and an instrumental variable generalized-method of moment (IV-GMM).

Here are the remaining sections of this study. The “Related prior studies and hypothesis development” section presents a review of the existing literature. A description of the research methodology and variables is given in the “Methodology and data” section. The “Estimation strategy” section presents the estimation strategy. The “Results and discussions” Section summarises the main findings, while the “Conclusions, policy implications, and future outlook” section concludes with policy implications.

## Related prior studies and hypothesis development

### Financial inclusion and CO_2_ emissions

The principle of financial inclusion is to ensure that financial products and services are accessible to all individuals and businesses. The World Bank acknowledged that fulfilling this principle would contribute to the accomplishment of the 17 United Nations Sustainable Development Goals (SDGs) (World Bank 2018). There are currently few studies on the relationship between financial inclusion and the environment, with conflicting theoretical perspectives and empirical results about how financial inclusion influences the quality of the environment (Le et al. 2020; Shahbaz et al. 2022; Said and Acheampong 2024). According to theoretical standpoint, financial inclusion could both negatively and positively influence the environment. By improving financial inclusion, individuals would be more likely to be able to access financial products and to use energy-consuming appliances such as refrigerators, automobiles, and coolers (Said et al. 2023; Le et al. 2020; Frankel and Romer 2017), leading to a higher level of CO_2_ emissions. In addition, through fostering inclusive financial systems, more economic activity occurs, which increases the demand for non-renewable energy sources and increases the amount of CO_2_ emitted into the atmosphere (Tao et al. 2022; Said and Acheampong 2024; Said 2024). However, according to the credit-innovation theory, financial inclusion has the potential to reduce CO_2_ emissions through green technology development. The development of green technologies is widely recognized as a key component of CO_2_ emission reduction (Du et al. 2019; Said and Acheampong 2024). Yang et al. (2024) support this point of view by stating that financial inclusion will reduce CO_2_ emissions by promoting green innovation, environmental procedures, and the use of clean energy.

As with theoretical ambiguity, empirical studies have also found inconsistent results regarding the impact of financial inclusion on CO_2_ emissions. As presented in Table 1, while some empirical studies suggest that financial inclusion increases CO_2_ emissions, other empirical studies suggest the opposite. For instance, Le et al. (2020) point out financial inclusion leads to higher CO_2_ emissions in the case of Asia. In addition, Mehmood (2022) indicates that financial inclusion exacerbates CO_2_ emissions in South Asia. Additionally, Ahmad et al. (2022) assess the influence of financial inclusion on environmental pollution in the ASEAN region from 2000 through 2019. The results indicate that financial inclusion brings out environmental degradation in the ASEAN region. In particular, they indicate that a 1% increase in financial inclusion results in a 0.42% increase in environmental degradation. Using a sample of Belt and Road Initiative (BRI) countries, Cai and Wei (2023) report that financial inclusion is associated with greater CO_2_ emissions. They find that 1% rise in financial inclusion increases CO_2_ emissions by 0.158%. The same conclusion is also reached by Mukalayi and Inglesi-Lotz (2023) who point out that financial inclusion contributes to the decline in environmental quality in Africa between 1990 and 2019. However, Du et al. (2022) contend that financial inclusion negatively impacts CO_2_ emissions, indicating that boosting financial inclusion is beneficial for the environment. Hussain et al. (2023), based on the pooled mean group estimation technique (PMG), explores the association between financial inclusion and CO_2_ emissions in a large sample of Asian economies. The results indicate that financial inclusion negatively impacts CO_2_ emissions over the long-term. Zhou et al. (2023) examine the effect of financial inclusion on carbon emissions using 30 Chinese provinces for the period 2011–2020. The empirical findings exhibit that financial inclusion results in a significant decrease in CO_2_ emissions, suggesting that financial access contributes to the environmental quality. Employing the AMG estimator and the Driscoll–Kraay panel regression approach, Prempeh et al. (2023) reveal that the development of the banking sector improves environmental quality in western African countries between 1990 and 2019. In a more recent study, Prempeh (2024) indicates that a higher level of financial development and the use of renewable energy are linked with lower levels of CO_2_ emissions in 10 West African countries. In addition, Said and Acheampong (2024) indicate that financial inclusion contributes significantly to decarbonization in the MENA region. According to the proposed relationship in the literature, we hold the following hypothesis:

- **H1.** Financial inclusion increases CO_2_ emissions in sub-Saharan Africa.

**Table 1:** Literature review-financial inclusion-CO_2_ emissions relationship

Authors	Period	Countries	Methodology	Results
Le et al. (2020)	2004–2014	31 Asian countries	Hoechle (2007) model	FI increases CO_2_ emissions
Mehmood (2022)	1990–2017	4 South Asian countries	ARDL approach	FI increases CO_2_ emissions
Ozturk and Ullah (2022)	2007–2019	42 BRI countries	GMM	FI increases CO_2_ emissions
Liu et al. (2022)	1995–2019	China	ARDL	FI decreases CO_2_ emissions
Shahbaz et al. (2022)	2011–2017	China	GLS	FI decreases CO_2_ emissions
Jebli and Hakimi (2023)	2004–2019	Top 10 technological countries	ARDL approach	FI increases CO_2_ emissions
Singh et al. (2023)	2008–2018	India	ARDL approach	FI increases CO_2_ emissions
Sharif et al. (2023)	1990–2019	ASEAN countries	ARDL approach	FI decreases CO_2_ emissions
Hussain et al. (2023)	2004–2020	102 countries	ARDL approach	No linear relationship
Mukalayi and Inglesi-Lotz (2023)	1990–2019	Sub-Saharan Africa	Fixed effects estimation technique	FI increases CO_2_ emissions
Zhou et al. (2023)	2011–2020	China	Two-way fixed effect panel estimation	FI decreases CO_2_ emissions
Prempeh et al. (2023)	1990–2019	11 West African states	AMG estimator	FI decreases CO_2_ emissions
Prempeh et al. (2023)	1990–2019	10 West African states	Driscoll-Kraay panel regression	FI decreases CO_2_ emissions
Said and Acheampong (2024)	2004–2019	MENA region	FMOLS, DOLS, and CCR	FI decreases CO_2_ emissions
Yang et al. (2024)	2011–2020	China	Spatial econometric models	FI decreases CO_2_ emissions

*FI*, financial inclusion; *ARDL*, autoregressive distributed lag model approach; *GMM*, generalized method of moments approach; *GLS*, generalized least squares approach; *FMOLS*, fully modified ordinary least square approach; *DOLS*, dynamic ordinary least square approach; *CCR*, canonical cointegrating regressions approach; *AMG*, augmented mean group approach

### Moderating role of renewable energy in financial inclusion-CO_2_ emissions nexus

It is indispensable to conduct research about renewable energy and the environment, as it provides policymakers with additional insight into achieving Sustainable Development Goals (SDGs), specifically Goals 7 (Affordable and Clean Energy) and 13 (Climate Action). As renewable energy has a lower carbon footprint, it may become an important tool for reducing CO_2_ emissions. Several countries are developing policies to raise the production of renewable energy. According Fahim et al. (2023a), ASEAN nations have increased their renewable energy share through investments in geothermal, solar wind, and hydroelectric sources. ASEAN countries have developed regulations and incentives to encourage businesses and individuals to use renewable energy in the future (Fahim et al. 2023b). Renewable energy sources have been expanded to speed up the transmission to an environmentally friendly economy.

Considering the importance of the renewable energy in environmental sustainability, a number of studies have explored the relationship between renewable energy and CO_2_ emissions. Overall, the empirical research on the renewable energy-CO_2_ emissions linkage can be categorised into several segments. The first one asserts that renewable energy has a detrimental influence on CO_2_ emissions. For instance, Aydoğan and Vardar (2020) investigate the impact of renewable energy on CO_2_ emissions for a large sample of emerging countries. The results point out that renewable energy decreases CO_2_ emissions. Wang et al. (2021) assess the relationship between renewable energy and CO_2_ emissions in ten developing nations. The empirical findings show that renewable energy consumption leads to a reduction in CO_2_ emissions. In addition, Mirziyoyeva and Salahodjaev (2022) reveal that renewable energy improves the environmental quality in top carbon-intense countries in the world. The results suggest that a 1% increase in renewable energy consumption leads to a reduction of 0.98% in CO_2_ emissions. Recently, Said and Acheampong (2024) examine the connection between renewable energy and environmental pollution in 11 MENA countries. The outcomes reveal that renewable energy reduces the carbon emissions in the region, thereby improving environmental quality.

The second segment of empirical studies argues that renewable energy consumption worsens environmental quality. Bulut (2017) analyzes the influence of renewable energy on Turkey’s environment. It has been shown that renewable energy use enhances CO_2_ emissions. By other words, renewable energy negatively impacts Turkish environmental quality. The same finding can also be found in Nguyen and Kakinaka (2019), who argue that renewable energy worsens environmental quality over time.

The last segment of studies reports that renewable energy exerts negligible impacts on CO_2_ emissions. Twumasi (2017), for instance, evaluates the influence of renewable energy on CO_2_ emissions in United States of America. Empirical findings indicate that renewable energy use wields no influence on CO_2_ emissions. Similarly, Boontome et al. (2017) contend that renewable energy has an inconsiderable influence on environmental quality in Thailand for the period 1971–2013. Nathaniel and Iheonu (2019) examine the association between renewable energy and environmental pollution in sub-Saharan African countries. The empirical findings indicate that renewable energy consumption has an unimportant influence on CO_2_ emissions. Similarly, Saidi and Omri (2020) point out that there is no link between CO_2_ emissions and renewable energy consumption in 15 major renewable energy nations from 1990 through 2014. Lastly, Pata et al. (2023) indicate that renewable energy does exert any impact on the environment in six ASEAN countries from 1995 to 2018. It is apparent from this review that the wide variety of empirical models used in these studies has resulted in inconclusive results.

Despite the direct role of renewable energy consumption in CO_2_ emissions, it can indirectly influence CO_2_ emissions by influencing the impact of financial inclusion on CO_2_ emissions. For instance, the use of renewable energy plays an important role in promoting financial inclusion (Işık et al. 2024). An environment-friendly energy policy attracts the attention of financial investors. In addition, as the financial sector improves, companies become more attractive to investing in energy, which in turn increases energy demand and creates more trust in the financial system (Işık et al. 2024). Many empirical studies have supported that renewable energy consumption matters in enhancing financial inclusion (Islam et al. 2013; Furuoka 2015; Anton and Nucu 2020). These discussions and evidence show that renewable energy can condition the effect of financial inclusion to influence CO_2_ emissions. It is, however, rare to find empirical evidence that demonstrate how renewable energy moderates the relationship between financial inclusion and CO_2_ emissions; hence, this insures additional investigation. Therefore, we formulate the second hypothesis below:

- **H2**: Renewable energy consumption moderates the relationship between financial inclusion and CO_2_ emissions.

### Moderating role of economic growth in financial inclusion-CO_2_ emissions nexus

Over the past few decades, the empirical link between economic growth and CO_2_ emissions has been heavily explored. This relationship is closely linked with testing the validity of the environmental Kuznets curve (EKC) hypothesis. Numerous present studies on this relationship have contended that the level of environmental pollution and economic growth follows the inverted U-shaped relationship. This inverted U-shaped linkage is identified as EKC in the literature. Since the 1990s, that relationship has been investigated after Grossman and Krueger (1995) presented empirical evidence suggesting that economic growth contributes to a gradually deteriorating environment in its early stages. However, after a certain threshold of economic growth, it contributes to an enhancement in the environmental conditions. Subsequent to this seminal study, numerous studies have investigated the link between growth and environmental pollution after Grossman and Krueger’s (1995) theory and demonstrate contradictory conclusions. For instance, Sapkota and Bastola (2017) test the validity EKC hypothesis between economic growth and environmental pollution in Latin American region over the period 1980–2010. Results indicate the presence of the EKC hypothesis in the region. Omri et al. (2015) also validates the existence of EKC hypothesis in MENA countries from 1990 to 2011. By contrast, numerous studies have different perspective regarding the link between economic growth and environmental pollution. Pablo-Romero and De Jesús (2016) test the EKC hypothesis between energy consumption and CO_2_ emissions in Latin America. The results indicate that EKC is not supported for the region. Acheampong (2019) indicates that EKC is not valid using data from 46 sub-Saharan African countries from 2000 to 2015. Similar results are also found in empirical studies such as Stern and Common (2001), Stern (2004), and Acheampong et al. (2020).

Although the evidence that economic growth is associated with CO_2_ emissions, economic growth indirectly affects CO_2_ emissions through its impact on financial inclusion. According to Sarma and Pais (2011), economic growth contributes to higher financial development, which in turn has an impact on financial inclusion. The authors indicate that financial inclusion is considerably affected by income, inequality, literacy, urbanization, and physical infrastructure in a country. In addition, Wang and Guan (2017) and Van et al. (2021) contend that an individual’s income and education play a crucial role in determining the level of financial inclusion in a country. Synthesizing the above evidence, we formulate the third hypothesis:

- **H3**: Economic growth moderates the link between financial inclusion and CO_2_ emissions.

### Gaps in the literature

Few studies have explored the influence of financial inclusion, economic growth, renewable energy, and CO_2_ emissions across different regions. The link between financial inclusion, economic growth, renewable energy use, and CO_2_ emissions varies from study to another. A difference in geographical location, economic development level, approach, selected variables, or econometrics techniques can also affect the results of research. To the author’s knowledge, none of these studies has explored this relationship in the case of sub-Saharan Africa. In the last two decades, Sub-Saharan countries have experienced improvements in terms of economic development, financial systems, and renewable energy consumption; therefore, examining this relationship would be worthwhile, as it would offer more insightful implications for these countries. Further, a number of recent studies investigated the role of finance in the renewable energy-CO_2_ emissions (see, e.g., Wang et al. 2022a); however, the roles of renewable energy and economic growth in moderating the influence of financial inclusion on environmental pollution is unknown. Aiming to address this issue, this paper explores the moderating roles of renewable energy and economic growth in the linkage between financial inclusion and environmental pollution in 23 nations in sub-Saharan Africa.

## Methodology and data

### Empirical model

This paper explores the moderating role of economic growth and renewable energy on financial inclusion-CO_2_ emissions nexus in a sample of 23 sub-Saharan countries from 2004 to 2019.^1^ To achieve this objective, this study considers three models. In the main effect model Eq. (1), CO_2_ emission is a function of financial inclusion (FI), economic growth (*GDPPC*), squared of economic growth (*GDPPC*)^2^, renewable energy consumption (*RE*), and industrialization (INDUS). As per existing literature (Shahbaz et al. 2022; Said and Acheampong, 2023), this study investigates the impact of financial inclusion on CO_2_ emissions using the empirical estimation shown in Eq. (1).

1 $$\begin{array}{c}{\text{ln CO}}_{2}{\mathrm{PC}}_{it}= {\theta }_{1}+{\beta }_{1}{\mathrm{lnRE}}_{it}\\ +{{\beta }_{2}{\mathrm{lnGDPPC}}_{it}}{+{{\beta }_{3}{{(\mathrm{lnGDPPC})}^{2}}_{it}+{\beta }_{4}{\mathrm{lnINDUS}}_{it}+\beta }_{5}{\mathrm{lnFI}}_{it}+{\mu }_{t}+\varepsilon }_{it}\end{array}$$

To probe the moderating roles of financial inclusion and economic growth and financial inclusion and renewable energy on CO_2_ emissions, Eqs. (2) and (3) extend Eq. (1) to consider the interaction terms of financial inclusion and economic growth (*lnFI* × *lnGDPPC*) and of financial inclusion and renewable energy (*lnFI* × *lnRE*). Equations (2) and (3) are written as follows:

2 $$\begin{array}{c}{\mathrm{ln }{\mathrm{CO}}_{2}\mathrm{PC}}_{it}= {\theta }_{1}+{\beta }_{1}{\mathrm{lnRE}}_{it}+{{\beta }_{2}{\mathrm{lnGDPPC}}_{it}}\\ +{{\beta }_{3}({{\mathrm{lnGDPPC})}^{2}}_{it}+{\beta }_{4}{\mathrm{lnINDUS}}_{it}+\beta }_{5}{\mathrm{lnFI}}_{it}+{\alpha }_{1}({\mathrm{lnFI}*\mathrm{lnGDPPC})}_{it}\\ +{\mu }_{t}+{\varepsilon }_{it}\end{array}$$

3 $${\mathrm{ln }{\mathrm{CO}}_{2}\mathrm{PC}}_{it}= {\theta }_{1}+{\beta }_{1}{\mathrm{lnRE}}_{it}+{{\beta }_{2}{\mathrm{lnGDPPC}}_{it}}{+{{\beta }_{3}({{\mathrm{lnGDPPC})}^{2}}_{it}+{\beta }_{4}{\mathrm{lnINDUS}}_{it}+\beta }_{5}{\mathrm{lnFI}}_{it}+{\alpha }_{2}({\mathrm{lnFI}*\mathrm{lnRE})}_{it}+{\mu }_{t}+\varepsilon }_{it}$$

where *i* = 1……..23 and *t* = 2004……0.2019; ${\beta }_{1}$…….${\beta }_{5}$ are the coefficients to be estimated; ${\alpha }_{1}$ and ${\alpha }_{2}$ capture the indirect effect of financial inclusion; ${\theta }_{1}$ represents the fixed country effect,${\mu }_{t}$ is time fixed effects, and $\upvarepsilon$ is the white noise.

Table 2 displays descriptive statistics between the variables. CO_2_ emissions per capita increase on average by 0.19% with a standard deviation of 0.65%. In addition, economic growth in sub-Saharan Africa averages 7.94%, while renewable energy usage averages 4.10%. Regarding financial inclusion indicators, ATMs in sub-Saharan Africa have an average number of 2.83%, while bank branches have an average number of 2.15%.

**Table 2:** Descriptive statistics (Logarithm form)

Variables	Mean	SD	Min	Max
lnCO2 PC	0.19	0.65	− 3.91	2.15
lnATM	2.83	2.99	− 3.22	4.50
lnBB	2.15	2.40	− 0.92	4.01
lnODCB	3.52	3.49	1.38	5.26
lnOLCB	3.15	2.96	− 0.24	4.90
lnRE	4.10	3.38	− 0.34	4.56
lnGDPPC	7.94	8.08	5.65	9.74
lnINDUS	3.23	2.29	2.14	4.13

Table 3 shows that there is a strong positive correlation between financial inclusion variables (lnATM, lnBB, and lnOLCB) and CO_2_ per capita (lnCO_2_PC). In addition, it has been reported a strong negative connection between renewable energy (lnRE) and lnCO_2_PC. Finally, the results show that the economic growth (lnGDPPC) is found to be positively and highly related with lnCO_2_PC.

**Table 3:** Correlations

	(1)	(2)	(3)	(4)	(5)	(6)	(7)	(8)
lnCO2 PC (1)	1.00							
lnATM (2)	**0.75**	1.00						
lnBB (3)	**0.58**	0.74	1.00					
lnODCB (4)	0.41	0.65	0.61	1.00				
lnOLCB (5)	**0.63**	0.77	0.52	0.80	1.00			
lnRE (6)	** − 0.77**	− 0.83	− 0.73	− 0.68	− 0.74	1.00		
lnGDPPC (7)	**0.79**	0.82	0.84	0.61	0.55	− 0.82	1.00	
lnINDUS (8)	− 0.02	− 0.01	− 0.20	− 0.11	− 0.06	− 0.07	− 0.03	1.00

Values in bold indicate a strong relationship between the two variables

### Description of variables

#### Financial inclusion index (lnFI)

To calculate a composite financial inclusion index (FI), four measures are considered: (1) number of ATMs per 10,000 adults, (2) number of commercial bank branches per 10,000 adults, (3) outstanding deposits from commercial banks (% of GDP), and (4) outstanding loans from commercial banks (% of GDP). Due to the different units and scales of these measures, it is necessary to transform them into normalized variables before aggregating them into a composite index (FI). Normalization using standardized *Z*-score approach is conducted as follows:

$$Z-\mathrm{score}=\frac{{X}_{i}-\overline{X}}{\alpha }$$

where ${X}_{i}$ denotes the raw score; $\overline{X }$ is the group average, and *α* is the standard deviation. Then, conduct a principal component analysis (PCA) on the normalized indicators.

#### CO_2_ emissions per capita (lnCO_2_ PC)

The variable CO_2_ emission is expressed in metric tons per capita. In the literature, CO_2_ emissions per capita have been widely used to assess environmental pollution (Boontome et al. 2017; Acheampong et al. 2020; Shahbaz et al. 2022; Said and Acheampong 2024). CO_2_ emissions are the primary cause of global warming.

#### Renewable energy (lnRE)

Switching to renewable energy is an important factor in improving the environment. It has been found that renewable energy consumption and CO_2_ emissions are negatively correlated regardless of the sample and methodology used (see, e.g., Charfeddine and Kahia 2019; Chen et al. 2019; Sharif et al. 2019; Yao et al. 2019), other evidence suggests that renewable energy has a positive or even neutral effect on CO_2_ emissions (Apergis et al. 2010; Menyah and Wolde-Rufael 2010; Saidi and Omri 2020).

#### Economic growth (lnGDPPC)

Economic growth is represented by real GDP per capita. By including per capita GDP and its square into the analysis, one can establish the EKC hypothesis, which assumes that CO_2_ emissions and per capita GDP are inversely related (Inverted U-shaped). Otherwise, it is U-shaped if GDP per capita is negative and significant, and its square term is positive and significant. The validity of the EKC hypothesis is contradictory (see, e.g., Stern 2004; Omri et al. 2015).

#### Industry (lnINDUS)

Industry affects CO_2_ emissions both positively and negatively. The impact of industrialization on CO_2_ emissions has been shown to be positive because it wields higher environmental pollution (Liu and Bae 2018). Alternatively, the development of the tertiary industry and the rationalization of the industrial structure lead to a reduction in environmental pollution (Du et al. 2019; Wang et al. 2022b).

## Estimation strategy

### Cross-section dependence test

Previous research shows that panel data can have heterogeneity and cross-section dependence (CD) issues. The effects of CD must be investigated as they can sometimes produce erroneous results (Danish et al. 2018). Hence, this study computes cross-sectional dependence using Pesaran’s CD test (Pesaran 2004). De Hoyos and Sarafidis (2006) suggested that disregarded common shocks can affect the cross-sectional dependence of cross-country panels. In other words, if cross-sectional dependence is present in data but not considered in the analysis, it will cause inconsistent standard errors (Driscoll and Kraay 1998). The results reported in Table 4 indicate that null hypothesis is not rejected in all tests, which confirms the residuals are cross-sectionally independent under fixed effect and random effect specifications.

**Table 4:** Results of the cross-sectional dependence tests

Test	Statistics	Prob	Statistics	Prob
Equation (1)	**Fixed effect**		**Random effect**	
Pesaran’s test	− 0.949	0.343	− 0.555	0.579
Friedman’s test	6.708	0.999	9.531	0.990
Equation (2)				
Pesaran’s test	− 0.985	0.325	− 0.477	0.634
Friedman’s test	6.708	0.999	10.398	0.982
Equation (3)				
Pesaran’s test	− 0.996	0.319	− 0.551	0.582
Friedman’s test	6.708	0.999	9.503	0.990

### Panel unit root test

Testing the stationarity of the variables is a crucial process in any study. The validation of cross-sectional independence led me to employ the unit root test recommended by Im et al. (2003). Table 5 shows that all variables have a unit root. As soon as the first difference between all variables is taken, stationarity is implemented, indicating that all variables are integrated of order 1. In order to estimate variables empirically, it is necessary to establish their stationary state.

**Table 5:** Results of panel unit root tests

Series	Level		First difference	
Variable	W-t-bar	*P*-value	W-t-bar	*P*-value
lnCO_2_ PC	0.901	0.816	− 5.396***	0.000
lnFI	− 0.586	0.279	− 5.047***	0.000
lnRE	1.620	0.947	− 5.070***	0.000
lnGDPPC	− 0.876	0.191	− 3.027***	0.001
lnINDUS	0.091	0.536	− 8.124***	0.000

^***^P < 0.01

### Panel co-integration test

Once all the series are stationary and integrated at order 1, it needs to be checked whether they cointegrate. This study uses the Pedroni panel co-integration test (Pedroni 2004).^2^ Pedroni test takes into account the heterogeneity through parameters that may differ between individuals. Under the null hypothesis, the test is based on the absence of co-integration. In contrast, under the alternative hypothesis, there is a co-integration relationship between the variables for each panel (Dinda and Coondoo 2006). Based on the results in Table 6, the test statistically rejects the null hypothesis of no co-integration and supports the alternative hypothesis regarding co-integration between variables in Eqs. (1), (2), and (3).

**Table 6:** Results of panel cointegration tests

	Cointegration test	Statistic	*P*-value
Equation (1)			
Pedroni test			
Ho: no cointegration–Ha: all panels are co-integrated			
1	Modified Phillips–Perron *t*	5.229***	0.000
2	Phillips–Perron *t*	− 2.771***	0.003
3	Augmented Dickey–Fuller *t*	− 2.669***	0.004
Equation (2)			
Pedroni test			
Ho: no cointegration– Ha: all panels are cointegrated			
1	Modified Phillips–Perron *t*	6.153***	0.000
2	Phillips–Perron *t*	− 3.295***	0.001
3	Augmented Dickey–Fuller *t*	− 2.812***	0.003
Equation (3)			
Pedroni test			
Ho: no cointegration–Ha: all panels are cointegrated			
1	Modified Phillips–Perron *t*	6.332***	0.000
2	Phillips–Perron *t*	− 2.356***	0.009
3	Augmented Dickey–Fuller *t*	− 2.272***	0.012

^***^P < 0.01

### Estimation of long-run coefficients

This study utilizes different estimation techniques to investigate the linkage between financial inclusion and environmental pollution. DOLS is used since it is a parametric estimator that addresses autocorrelation (Liguo et al. 2022). However, FMOLS approach takes into consideration the heterogeneity in the cointegration link. Additionally, it rectifies the endogeneity of the explanatory indicators and the serial link inherent in the dynamic panels (Pedroni 2001). CCR employs a stationary conversion approach to reduce the relation between stochastic regressor errors and cointegration equations (Christiansen 2015). To check the robustness of the outcomes in presence of possible endogeneity between financial inclusion and environmental quality, this paper also utilizes the instrumental variable generalized method of moment (IV-GMM). The purpose of this study is to examine the effect of financial inclusion on CO_2_ emissions, so it is essential to have an instrument for financial inclusion index. This study employs the lags of the financial inclusion index as the instruments for financial inclusion index.^3^ To test the validity of the instruments, Kleibergen-Paap rk LM is used to examine whether the endogenous regressor is well identified by the instruments. Under the null hypothesis, the model is under-identified. However, the rejection of the null indicates that the model is identified. This paper also uses Cragg-Donald Wald *F* statistic test to examine whether the excluded instruments are correlated with the endogenous regressors. Under the null hypothesis, the model is weak. In contrast, the rejection of the null indicates the model is well-specified. Finally, Hansen *J* test of over-identifying restrictions is used. The null hypothesis of this test indicates that the instruments are valid instruments, i.e., uncorrelated with the error term. However, the rejection of this test indicates that the instruments are not valid, i.e., correlated with the error term.

## Results and discussions

Table 7 reports the panel results of the influence of financial inclusion on CO_2_ emissions. The empirical findings indicate that renewable energy is negatively and significantly correlated with CO_2_ emissions at a 1% significance level in all specifications, reflecting the potentially favorable influence of renewable energy on the environment. A 1% increase in renewable energy leads to a reduction in CO_2_ emissions per capita within a range of 0.394–0.414%. This outcome agrees with the previous literature, which indicates that renewable energy decreases CO_2_ emissions (see, e.g., Charfeddine and Kahia 2019; Abbasi et al. 2021; Said et al. 2022; Said and Acheampong 2024). However, the results are not in line with Twumasi (2017), Saidi and Omri (2020), Pata et al. (2023), and Mukhtarov (2024), which show that renewable energy consumption exerts insignificant influence on CO_2_ emissions. The coefficients of GDP per capita and its square term, respectively, have significant positive and negative effects on CO_2_ emissions. This indicates that income has an inverted-U shape relationship with pollution and, thus, supports environmental Kuznets curve (EKC) hypothesis for sub-Saharan Africa. It implies that the level of per capita CO_2_ emissions initially increases with per capita GDP and, after a certain level of growth, any decrease in per-capita GDP increases the per-capita CO_2_ emissions. This outcome does not agree with the work of Acheampong (2019) that shows that the EKC postulate is not supported in sub-Saharan Africa. However, the results are in line with the empirical work of Sapkota and Bastola (2017) which demonstrates that the EKC hypothesis is valid in Latin America. Furthermore, industry exerts a significant positive effect on CO_2_ emissions in all specifications. A 1% increase in industry increases per capita CO_2_ emissions within a range of 0.282–0.347%. This outcome agrees with the works of Le et al. (2020) and Dong et al. (2022) which indicate that industrialization contributes to higher environmental pollution. The results further indicate that financial inclusion positively related with CO_2_ emissions in all regressions. A 1% rise in the financial inclusion index increases CO_2_ emissions per capita within a range of 0.104–0.121%. The results support the empirical studies of Le et al. (2020), Singh et al. (2023), Fareed et al. (2022), and Said et al. (2023) which indicate that financial inclusion worsens the environmental quality. However, the results do not align with the findings of Shahbaz et al. (2022), Du et al. (2022), and Said and Acheampong (2024) which contend that financial inclusion has a beneficial impact on the environment.

**Table 7:** Financial inclusion and CO_2_ emissions

	Model 1	Model 2	Model 3	Model 4
Variable	DOLS	FMOLS	CCR	IV-GMM
lnRE	− 0.406*** (0.109)	− 0.394*** (0.062)	− 0.394*** (0.064)	− 0.414*** (0.051)
lnGDPPC	3.712*** (0.886)	3.781*** (0.492)	3.782*** (0.510)	3.551*** (0.347)
lnGDPPC^2^	− 0.185*** (0.062)	− 0.189*** (0.035)	− 0.189*** (0.036)	− 0.177*** (0.024)
lnINDUS	0.347** (0.138)	0.283*** (0.078)	0.282*** (0.080)	0.344*** (0.046)
lnFI	0.114** (0.057)	0.104*** (0.032)	0.104*** (0.033)	0.121*** (0.036)
Constant	− 17.471*** (2.839)	− 17.591*** (1.583)	− 17.594*** (1.636)	− 16.760*** (1.125)
Kleibergen-Paap rk LM statistic-*P* value				0.000
Cragg-Donald Wald *F* statistic-*P* value				1742.749
Hansen *J* statistic-*P* value				0.594
Number of observations	342	344	344	301
Number of countries	23	23	23	23

Under-identification is the Kleibergen–Paap rk test; weak identification test is Cragg-Donald Wald *F* statistic; over-identification test is the Hansen *J* statistic. **P < 0.05, ***P < 0.01

Lastly, the Hansen *J* test does not reject the over-identifying restrictions, affirming the validity of the instruments. Furthermore, the Kleibergen–Paap rk test and Cragg-Donald Wald *F* test confirm that these specifications are properly identified and do not suffer from under- and weak-identification problems.

Table 8 reports the panel results of the influence of financial inclusion on CO_2_ emissions in the presence of the interaction term of financial inclusion and economic growth. The results are at large similar to those presented in Table 7. Renewable energy decreases CO_2_ emissions per capita, industry increases CO_2_ emissions, and EKC hypothesis is valid between economic growth and environmental pollution. On the contrary, financial inclusion exerts a significant negative effect on CO_2_ emissions. As shown in Table 8, the interaction term enters with a positive sign, significant at the 5% level, suggesting a complementary relationship between finance and economic growth to worsen environmental quality. Financial inclusion could promote strong economic growth and encourage industrial activities, which leads to increasing CO_2_ emissions. A 1% increase in FI* GDPPC increased CO_2_ emissions with a range of 0.133–0.194%.

**Table 8:** Interaction effect of financial inclusion and economic growth on CO_2_ emissions

	Model 1	Model 2	Model 3	Model 4
Variable	DOLS	FMOLS	CCR	IV-GMM
lnRE	− 0.394*** (0.066)	− 0.383*** (0.045)	− 0.383*** (0.046)	− 0.412*** (0.044)
lnGDPPC	4.532*** (0.561)	4.603*** (0.375)	4.602*** (0.387)	4.099*** (0.421)
lnGDPPC^2^	− 0.244*** (0.040)	− 0.249*** (0.026)	− 0.249*** (0.027)	− 0.216*** (0.030)
lnINDUS	0.324*** (0.084)	0.299*** (0.057)	0.299*** (0.058)	0.342*** (0.043)
lnFI	− 1.298*** (0.288)	− 1.266*** (0.193)	− 1.267*** (0.199)	− 0.854* (0.459)
Ln(FI *GDPPC)	0.194*** (0.039)	0.188*** (0.026)	0.188*** (0.027)	0.133** (0.062)
Constant	− 20.354*** (1.818)	− 20.585*** (1.217)	− 20.585*** (1.257)	− 18.686*** (1.409)
Kleibergen-Paap rk LM statistic-*P* value				0.000
Cragg-Donald Wald *F* statistic-*P* value				41.042
Hansen *J* statistic-*P* value				0.536
Number of observations	342	344	344	301
Number of countries	23	23	23	23

Under-identification is the Kleibergen–Paap rk test; weak identification test is Cragg-Donald Wald *F* statistic; over-identification test is the Hansen *J* statistic. *P < 0.10, **P < 0.05, ***P < 0.01

Table 9 provides similar results to Table 7 in relation to renewable energy, economic growth, industry, and financial inclusion impact on CO_2_ emissions. It is observed from Table 9 that the interaction term of financial inclusion and renewable energy wields a negative influence on CO_2_ emissions. This result suggests that financial inclusion complements renewable energy to reduce the CO_2_ emissions. Hence, financial inclusion assures efficacy in renewable energy, thereby lessening CO_2_ emissions. A 1% increase in FI* RE significantly reduced CO_2_ emissions within a range of 0.282–0.336%.

**Table 9:** Interaction effect of financial inclusion and renewable energy on CO_2_ emissions

	Model 1	Model 2	Model 3	Model 4
Variable	DOLS	FMOLS	CCR	IV-GMM
lnRE	− 0.284*** (0.094)	− 0.275*** (0.041)	− 0.275*** (0.042)	− 0.286*** (0.050)
lnGDPPC	4.229*** (0.727)	4.278*** (0.317)	4.277*** (0.328)	4.147*** (0.425)
lnGDPPC^2^	− 0.224*** (0.051)	− 0.227*** (0.022)	− 0.227*** (0.023)	− 0.221*** (0.030)
lnINDUS	0.404*** (0.113)	0.353*** (0.050)	0.352*** (0.051)	0.423*** (0.053)
lnFI	1.258*** (0.293)	1.240*** (0.130)	1.240*** (0.134)	1.447*** (0.373)
Ln(FI *RE)	− 0.288*** (0.073)	− 0.283*** (0.032)	− 0.282*** (0.033)	− 0.336*** (0.092)
Constant	− 19.965*** (2.375)	− 20.030*** (1.042)	− 20.027*** (1.074)	− 19.641*** (1.496)
Kleibergen-Paap rk LM statistic-*P* value				0.000
Cragg-Donald Wald *F* statistic-*P* value				41.907
Hansen *J* statistic-*P* value				0.359
Number of observations	342	344	344	301
Number of countries	23	23	23	23

under-identification is the Kleibergen–Paap rk test; weak identification test is Cragg-Donald Wald *F* statistic; over-identification test is the Hansen *J* statistic. ***P < 0.01

Finally, the Hansen *J* test does not reject the over-identifying restrictions affirms the validity of the instruments. In addition, The Kleibergen–Paap rk test and Cragg-Donald Wald *F* test suggest that this regression is well specified.

## Conclusions, policy implications, and future outlook

The present study may provide the first empirical analysis of the association between financial inclusion, economic growth, renewable energy, and CO_2_ emissions for 23 sub-Saharan African economies over the period 2004–2019. As estimation approaches, DOLS, FMOLS, and CCR are employed, as well as the IV-GMM estimator to determine the reliability of the results. The results of this research can be outlined in the following manner:

First, the findings demonstrate that financial inclusion exerts a significant positive effect on CO_2_ emissions in sub-Saharan Africa over the investigated period. The results indicate that a 1% increase in the financial inclusion index increases CO_2_ emissions per capita within a range of 0.104–0.121%. On the contrary, the results indicate that renewable energy has a significant negative impact on CO_2_ emissions. Specifically, a 1% increase in renewable energy use can reduce CO_2_ emissions within a range of 0.394–0.414%. The results also validate the EKC hypothesis in sub-Saharan Africa.

Second, the results indicate that the financial inclusion moderates economic growth to increase the CO_2_ emissions. However, our empirical findings show that financial inclusion moderates renewable energy to reduce CO_2_ emissions in the region. While this paper establishes that financial inclusion directly deteriorates environmental quality, it indirectly improves the quality of the environment by promoting renewable energy use.

These findings have three main implications for policies for attaining SDG 7 in sub-Saharan Africa. This study calls on policymakers to enact measures that warrant the stability and effectiveness of the financial system. Finding the adverse environmental influence of financial inclusion definitely does not mean lowering financial inclusion. Instead, policymakers should strive to improve the environmental impact of financial inclusion. Consistent with the recommendation of Le et al. (2020) and Said et al. (2023), for financial inclusion to drive SDG 7, policy makers should support access to financial services initiatives consistent with environmental policies in region-wide countries. Secondly, financial inclusion indirectly increases CO_2_ emissions by spurring economic growth in sub-Saharan nations. It is important for policy makers to encourage investment in sustainable economic sectors. Finally, financial inclusion indirectly reduces CO_2_ emissions by promoting renewable energy in the region; policymakers should promote renewable energy use through policy support, public awareness, and investments in research and development. Moreover, tax incentives should be offered to encourage the use of renewable energy in these nations. Thus, this will spur the production of clean and modern energy (Mentel et al. 2022).

Despite contributing to the relevant research area, the current work has some limitations. Firstly, this study uses CO_2_ emissions as the only proxy for the environmental pollution. Nevertheless, additional proxies of environmental pollution, such as sulfur dioxide (SO2) and nitrogen dioxide (NO2), can be used to ensure robustness. Furthermore, a narrow definition of financial inclusion was constructed in this study. Various aspects should be included to create a financial inclusion index, such as credit, savings, payments, and insurance.

While this study makes a valuable contribution, some further research is still needed. There are numerous ways to expand this research. Future research could look at the relationship between economic growth, financial inclusion, and CO_2_ emissions in other developing regions. Second, future studies can explore the impact of financial inclusion, economic growth, and renewable energy on ecological footprint, which generally describes environmental degradation. Lastly, a comparative study that concentrates on the degree of a nation’s economic development may help to clarify the impact of financial accessibility and economic expansion on CO_2_ emissions.

## Data Availability

Data are available upon request.

## Appendix

See Table 10

**Table 10:** Sample composition

Country name	Observation period
Angola	[2004–2019]
Botswana	[2004–2019]
Burundi	[2004–2016]
Cabo Verde	[2004–2019]
Cameroon	[2004–2019]
Comoros	[2004–2019]
Eswatini	[2004–2019]
Ghana	[2008–2019]
Guinea	[2004–2019]
Guinea-Bissau	[2010–2019]
Kenya	[2004–2019]
Lesotho	[2004–2019]
Madagascar	[2004–2019]
Mauritius	[2004–2019]
Mozambique	[2004–2019]
Namibia	[2006–2019]
Nigeria	[2004–2019]
Rwanda	[2004–2019]
Seychelles	[2004–2019]
South Africa	[2004–2019]
Uganda	[2004–2019]
Zambia	[2004–2019]
Zimbabwe	[2009–2019]

## Footnotes

1 See Table 10 in the Appendix for the list of sub-Saharan African countries included in the analysis.

2 Pesaran’s (2004) CD test is chosen as it is more appropriate for unbalanced panels.

3 This study uses the first and second lags of financial inclusion as instruments of financial inclusion index.
